# Genetic diversity of alpha and betacoronaviruses in cave and temple-roosting bats in Vientiane Province, Lao PDR

**DOI:** 10.1371/journal.pone.0341737

**Published:** 2026-01-30

**Authors:** Illich Manfred Mombo, Novy Charel Bobouaka Bonguili, Nicola Gasperini, Arisa Chandarak, Thida Xaiyaphoumi, Pauline Brault, Oceane Rieu, Phimpha Paboriboune, Eric Leroy, Fabien Roch Niama, Sabrina Locatelli

**Affiliations:** 1 Centre interdisciplinaire de Recherches Médicales de Franceville, Franceville, Gabon; 2 UMR TransVIHMI, Université de Montpellier – IRD – Inserm, Montpellier, France; 3 Faculté des Sciences et Techniques, Université Marien Ngouabi, Brazzaville, République du Congo; 4 Laboratoire National de Santé Publique, Brazzaville, République du Congo; 5 Independent Researchers-IRD associated, Vientiane, Lao PDR; 6 Faculty of Pharmacy, University of Health Sciences, Vientiane, Lao PDR; 7 UMR MIVEGEC, University of Montpellier, IRD, CNRS, Montpellier, France; 8 Centre d’Infectiologie Lao-Christophe Mérieux (CILM), Vientiane, Laos; UNAM: Universidad Nacional Autonoma de Mexico, MEXICO

## Abstract

The emergence of MERS-CoV, SARS-CoV-1, and SARS-CoV-2 highlights the significant public health and economic threats posed by coronaviruses. In Lao PDR, SARS-CoV-2-related bat coronaviruses capable of binding to human ACE2 receptors have been found in northern regions, but little is known about coronavirus diversity in anthropized environments like temples. This study investigated coronavirus circulation, diversity, and prevalence in bats from caves and temples in Vientiane Province, Lao PDR. A total of 648 guano samples (505 *Chaerephon plicatus*, 100 *Hipposideros* spp., 43 *Taphozous* spp.) were collected between December 2022 and June 2023 and screened using pan-coronavirus RT-PCR approach. The overall positivity rate was 17.28%, significantly higher in caves (18.8%) than temples (4.41%) (p = 0.003). *C. plicatus* showed the highest positivity rate (21.38%), followed by *Hipposideros* spp. 4%, while *Taphozous* spp. were negative. Phylogenetic analysis revealed diverse coronavirus lineages within Alphacoronavirus (80.4%) and Betacoronavirus (19.6%) genera. Although none were closely related to known human pathogens, coronaviruses of *Decacovirus* genus related to Chinese bat viruses and *Pedacovirus* genus similar to porcine epidemic diarrhea virus (PEDV) were detected. Unclassified betacoronaviruses identified were also related to viruses from *C. plicatus* in Thailand. This study provides valuable insights into coronavirus circulation in both natural and anthropized environments. The detection of PEDV-like viruses underlines the need for continued surveillance at the human-bat interface, where activities like guano harvesting and temple visits increase contacts. Further genomic and functional studies would enhance our understanding of their evolutionary relationships and potential for further cross-species transmission.

## Introduction

Coronaviruses (CoVs) represent a diverse group of enveloped, positive single-strand RNA viruses belonging to the genera *Alphacoronavirus*, *Betacoronavirus*, *Gammacoronavirus*, and *Deltacoronavirus* of the subfamily *Orthocoronavirinae* in *Coronaviridae* family (ICTV, 2023). Alpha-CoVs and beta-CoVs primarily infect mammals, while gamma-CoVs and delta-CoVs typically infect birds and fish, though they retain the potential to infect mammals [1]. Currently, seven CoVs are known to infect humans: four that generally cause mild respiratory infections (HCoV-229E, HCoV-HKU1, HCoV-OC43, and HCoV-NL63) and three highly pathogenic viruses—Middle East Respiratory Syndrome (MERS)-CoV, Severe Acute Respiratory Syndrome (SARS)-CoV-1, and SARS-CoV-2—that cause severe lower respiratory tract infections with a higher probability of developing acute respiratory distress syndrome and extrapulmonary manifestations [2].

CoVs possess the largest RNA viral genomes, ranging from approximately 27–32 kb. This extensive genome is subject to high error rates during replication, with elevated frequencies of recombination and mutation, conferring on CoVs the potential to adapt to new host species [3,4]. Combined with their wide diversity of hosts—including pets, livestock, poultry, wildlife, and humans—CoVs demonstrate significant potential for interspecies transmission, positioning them as viruses of particular concern for emerging infectious diseases [5].

In recent decades, three novel CoVs with epidemic or pandemic potential have emerged, causing severe respiratory diseases: SARS-CoV-1 in China (2002−2003), MERS-CoV in the Arabian Peninsula (2012), and SARS-CoV-2, first detected in Wuhan, China (2019) [6–8]. Multiple studies based on genetic evolution, pathogenesis mechanisms, and receptor binding characteristics have suggested that these CoVs originated in bats [9,10]. However, spillover to humans typically results from zoonotic transmission requiring intermediate hosts, such as palm civets (*Paguma larvata*) or common raccoon-dogs (*Nyctereutes procyonoides*) for SARS-CoV-1, and dromedary camels (*Camelus dromedarius*) for MERS-CoV [6,11]. For SARS-CoV-2, the intermediate host has not been definitively confirmed, though various candidates have been proposed such as ferrets (*Mustela putorius furo*), mink (*Neonvison vison*), raccoon dogs (*N. procyonoides*) and pangolins (*Manis spp*) [12].

Bats, comprising approximately 1,400 known species and constituting over 20% of all mammalian diversity, are increasingly recognized as natural reservoirs of numerous zoonotic viruses, including Ebolavirus, Marburgvirus, Nipah virus, and coronaviruses [13]. Their unique immune system effectively protects them against disease development, enabling them to maintain viruses without manifesting evident clinical signs of infection [14,15]. This viral tolerance, combined with their high population densities, gregarious roosting behavior, and extensive geographic distributions, positions bats as critical hosts in viral ecology and emergence [16].

Southeast Asia, with its exceptional bat diversity and habitat complexity, represents a particularly important region for bat-associated viruses. Surveillance efforts across the region have revealed considerable coronavirus diversity in bat populations. In Thailand, multiple coronavirus lineages in bats, including group C betacoronaviruses has been identified in bat guano used as fertilizer [17]. In Vietnam, novel coronaviruses has been detected in bats from the Mekong Delta region [18]. Similarly, surveillance in Cambodia led to the discovery of a novel SARS-CoV-2 related coronavirus in *Rhinolophus* bats [19]. A comprehensive study across southern China and parts of Southeast Asia revealed extensive evolutionary history and cross-species transmission patterns of bat coronaviruses, identifying several hotspots of coronavirus diversity, including areas bordering Laos [20].

In Laos specifically, recent surveys have documented over 90 bat species, with numerous caves, karst formations, and human structures serving as roosting sites [21]. The interface between humans and bats is intensified by activities such as guano harvesting, cave tourism, and religious practices at temples where bats roost, creating potential pathways for viral spillover [17,22]. Bat-borne coronaviruses were identified in northern Laos (Fueng and Meth districts of Vientiane Province) with remarkable similarity to SARS-CoV-2 [23]. These viruses, named BANAL-52, BANAL-103, and BANAL-236, were isolated from *Rhinolophus* bats and shared 96.8% genome identity with SARS-CoV-2. Most significantly, they possessed identical receptor binding domains capable of binding to human ACE2 receptors with the same efficiency as the early pandemic SARS-CoV-2 strain, suggesting that viruses with direct zoonotic potential were circulating in Laotian bats prior to the emergence of SARS-CoV-2.

Earlier surveillance in bats from Laos documented both alpha and betacoronaviruses, with a 6.5% overall prevalence across 21 bat species [24]. They identified several distinctive viral lineages not previously detected in neighboring countries, suggesting the presence of unique evolutionary lineages in Laotian bat populations. Additionally, the detection coronaviruses in rodents from Laos, indicated that multiple mammalian taxa harbor these viruses, potentially creating complex multi-host systems for viral evolution and transmission [25].

In a broader regional context, extensive coronavirus diversity has been documented in Southeast Asian bats, with Laos hosting several unique viral lineages [20]. Their phylogeographic analysis indicated substantial viral exchange across national borders, particularly between southern China, northern Vietnam, and northern Laos, suggesting that these areas represent a continuous ecological zone for coronavirus circulation.

Despite these important discoveries, significant knowledge gaps remain regarding coronavirus distribution and diversity across different bat roosting environments in Laos. In particular, the role of anthropized environments such as temples in viral ecology remains virtually unexplored. Temples represent unique settings where bats and humans coexist in close proximity, creating distinctive interfaces for potential viral transmission that differ substantially from natural cave settings. These religious structures offer protected spaces where bats can establish colonies, while simultaneously exposing them to human activities such as religious ceremonies, tourism, and maintenance work.

Despite their ecological and epidemiological significance, temples have been largely overlooked in coronavirus surveillance efforts, with most previous studies focusing on natural cave systems, human dwellings, or agricultural settings. Although study a in Thailand has included some temple locations within broader surveillance efforts, no studies have systematically compared coronavirus prevalence and diversity between temple-roosting and cave-dwelling bat populations [26]. This knowledge gap is particularly relevant in Laos, where temples serve as important cultural centers and frequently harbor significant bat populations.

The frequency of emerging zoonotic diseases is increasing due to major drivers such as environmental changes, demographic shifts, and ecological transformations leading to increased contact between humans and wildlife [27,28]. To prevent future outbreaks, active surveillance in wildlife, particularly in bats—recognized as the mammalian group harboring the greatest diversity of CoVs—is essential. Understanding the nature of bat-hosted CoVs, their association with specific bat species, and their genetic diversity is crucial for predicting potential host transitions and disease outbreaks [29,30].

Given these considerations, this study aims to investigate the circulation patterns, genetic diversity, and prevalence of coronaviruses in bat populations inhabiting caves and temples in Vientiane Province, Lao PDR. By specifically targeting both temple and cave environments, our study represents the first comparative analysis of coronavirus ecology across these distinct habitat types in Lao PDR, providing novel insights into how anthropized religious settings may influence viral diversity and prevalence in bat populations. This research seeks to characterize the coronavirus landscape at the human-wildlife interface, establish baseline data for long-term monitoring, and contribute to global efforts in identifying potential zoonotic threats before they emerge in human populations.

## Materials and methods

### Study sites and samples collection

**Fig 1:**
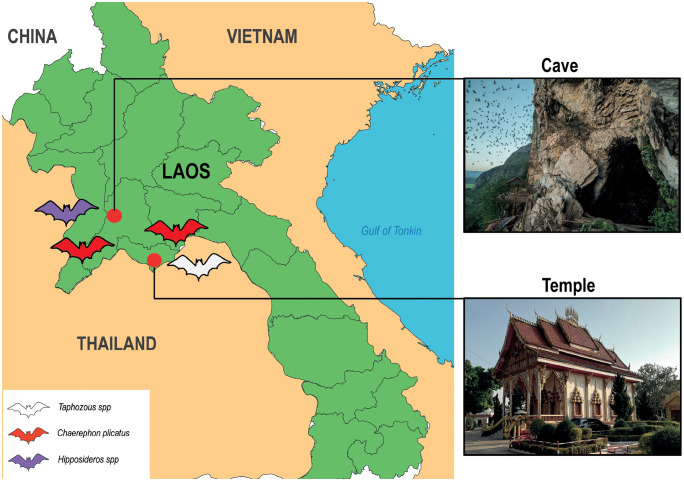
Geographical location of study sites.

The study was conducted from December 2022 through June 2023 in Vientiane province, Lao PDR (Fig 1). Sample sites included both a natural ecosystem represented by the Than Pha Luang cave and three temples—Vat Sisaket (VTESSK), Vat Phon Keng (VTEPNK) and Vat Phosainyalam (VTEPHS)—where humans and bats exhibit spatial overlap. Within temple environments, human-bat interactions were primarily characterized by spatial overlap, where bats roosted in temple structures such as rafters and eaves, while humans conducted daily religious activities in the spaces below. Indirect contact occurred when human activities including cleaning, maintenance, and ceremonies took place in areas recently occupied by bats. Temporal overlap was observed as peak human activity during morning prayers and evening ceremonies coincided with bat departure and return times. Temple staff and visitors occasionally encountered bats during cleaning activities or found individuals on the ground, though no evidence of intentional feeding, capturing, or regular handling of bats was observed. The relationship was primarily characterized by spatial coexistence rather than direct physical interaction.

Temples sampling sites distances were as follows: VTESSK – VTEPNK: 3.5 Km; VTESSK- VTEPHS: 19 Km; VTEPNK- VTEPHS: 18 Km. The maximum distance between sites was 90 Km between the Than Pha Luang cave and the three temples in Vientiane capital (Fig 1). Given that bats have documented foraging ranges of 27–217 km [31], potential movement of individuals between sites cannot be excluded.

Temples were selected based on confirmed presence of established bat roosts with populations of 50–100 individuals observed during preliminary surveys. Human activity levels varied among sites, with VTESSK receiving the highest number of daily visitors due to its tourist attraction status and regular morning and evening ceremonies, similar to the other two selected temples. Sites were chosen based on accessibility and permission availability, allowing sampling to be conducted with minimal disturbance to both religious activities and bat colonies. The temples represented similar architectural styles and roost microenvironments, ranging from open rafters to enclosed spaces.

Than Pha Luang cave was selected as a natural reference site with minimal human disturbance, hosting large colonies with an estimated population of up to 1 million individuals. Population estimates were based on 40-minute dusk emergence observations, with an average of 10,000–15,000 bats per minute and peak populations occurring in August. The site experiences regular human presence, with guano collectors harvesting the material for use as agricultural fertilizer.

Non-invasive sampling was performed to collect bat guano using sterile aluminum foils (1m length and 0.6 width) deposited under roosting sites before sunset. Early the following morning, fresh guano pellets were collected with sterile disposable picks and individually placed in a 96-wells plates of 0.2 ml containing 100 µl of homemade RNA*later*. Samples were transported under cold chain conditions and stored at −80°C at the laboratory at the faculty of Pharmacology of the University of Health Sciences in Vientiane then sent to Institut de Recherche pour le Développement in France for subsequent molecular analyses.

To investigate the dynamics of coronavirus infection in bats, longitudinal monitoring of the cave and temples was carried out throughout the collection period. Guano samples were collected every two months during the seven-month sampling period, resulting in three collection time points (December 2022, February 2023, and June 2023). The map was download freely on https://simplemaps.com/gis/country/la, and edited using QGIS software version 3.42.1 Münster.

### Nucleic acids extraction

The guano-RNAlater mixture was first homogenized thoroughly by vortexing for 30 seconds, then centrifuged at 5,000 g for 10 minutes at 4°C. Nucleic acid extraction was performed using 100 μL of the resulting supernatant with the NucleoSpin 96 Virus Core Kit (Macherey-Nagel, Düren, Germany), following the manufacturer’s instructions. The extracted nucleic acids were stored at −80°C until further analysis. After extraction, 100 μL of fresh RNA*later* was added to each well containing the guano pellet to preserve the remaining sample for potential future extractions.

### cDNA synthesis and coronaviruses RNA detection

The extracted RNA was reverse-transcribed into cDNA using Superscript IV Reverse Transcriptase (Invitrogen, Illkirch, France) in a final volume of 20 µL, as previously described [32,33]. The reaction was performed in two steps. First, 10 µL of RNA was combined with 1 μL dNTPs (10 mM), 1 μL random hexamer primers (50 ng/μL), and 1 μL DNase-free water, then incubated at 65°C for 5 minutes followed by 1 minute on ice to denature RNA secondary structures. Second, a mixture containing 4 µL of 5X Superscript IV Buffer, 1 µL of DTT (100 mM), 0.5 µL of RNAse Out, 0.5 µL of enzyme, and 1 µL of DNase-free water, was added to the previous mix. The reverse transcription program consisted of 23°C for 10 minutes (primer annealing), 50–55°C for 10 minutes (cDNA synthesis), and 80°C for 10 minutes (enzyme inactivation).

Samples were then screened for coronavirus RNA using a nested PCR approach with a set of degenerate primers targeting a 440 bp fragment of the highly conserved RNA-dependent RNA polymerase (RdRp) gene region found in all known coronaviruses (positions 14375–14816 of Porcine Enteric Diarrhea Virus, accession number KR265805). The primers used were: Pan-CoV-F1: 5’-GGKTGGGAYTAYCCKAARTG-3’ and Pan-CoV-R1: 3’-TGYTGTSWRCARAAYTCRTG-5’; Pan-CoV-F2: 5’-GGTTGGGACTATCCTAAGTGTGA-3’, Pan-CoV-R2: 5’-CCATCATCAGATAGAATCATCAT-3’ [34]. Both PCR rounds were performed using Platinum Taq DNA Polymerase (Invitrogen, Carlsbad, CA, USA). For the first round, the reaction mixture consisted of 5 µL of cDNA, 2.5 µL of 10X reaction buffer, 0.75 µL of MgCl2 (50 mM), 0.5 µL of dNTPs (10 mM), 1 µL of each primer (10 µM), and 0.1 µL of Platinum Taq Enzyme, and nuclease-free water to a final volume of 25 μL. The second round was performed with the same components and volumes, using 5 μL of the first-round PCR product as template. The thermocycling conditions for both rounds were set up as previously described [32,33]. SARS-CoV 2 RNA extracted from the nasal swab of a dog tested positive has been used as a positive control. Amplicons were visualized on a 1.5% agarose gel stained with ethidium bromide after electrophoresis at 100V for 30 minutes. Positive samples were sent to Eurofins Genomics GmbH (Ebersberg, Germany) for Sanger sequencing in both directions.

### Bat species identification

Bat species identification was performed for all samples to characterize the diversity of bat species inhabiting caves and temples. A 400 bp fragment of the 12S rRNA mitochondrial gene was amplified by PCR using the Platinum Taq DNA polymerase (Invitrogen, Carlsbad, CA, USA) as previously described [35]. The 25 μL reaction mixture consisted of 5 µL of extracted DNA, 2.5 µL of 10x reaction Buffer, 0.75 µL of MgCl2 (50 mM), 0.5 µL of dNTPs (10 mM), 1.5 µL of each primer (10 μM) (12S-L1091: 5’-AAAAAGCTTCAAACTGGGATTAGATACCCCACTAT-3’ and 12S-H1478: 5’-TGACTGCAGAGGGTGACGGGCGGTGTGT-3’), 1 µL of bovine serum albumin (BSA) (1 µg/µL), 0.1 µL of enzyme (5 U/μL), and nuclease-free water to reach the final volume.

The PCR cycling conditions were set up as previously described [35]. Amplicons were visualized on a 1.5% agarose gel after electrophoresis and purified prior to sequencing. Purified amplicons were sent to Eurofins Genomics (Germany) for bidirectional Sanger sequencing. Bat species were identified through the Basic Local Alignment Search Tool (BLAST, https://blast.ncbi.nlm.nih.gov/) by comparing the obtained 400 bp sequences of the 12S mitochondrial gene with reference sequences available in the GenBank database. Species identification was considered reliable when sequence similarity was ≥ 98% with a reference sequence from a voucher specimen.

### Phylogenetic analyses

The raw RdRp sequences obtained in this study were assembled and edited using the Seqman program implemented in LaserGen 7 (DNAstar, Madison, WI, USA). Consensus sequences were compared with a curated dataset of reference sequences representing all known coronavirus genera retrieved from GenBank using BLAST. Multiple sequence alignments were performed using the ClustalW algorithm implemented in MEGA 11 software (version 11.0.13). Evolutionary models were tested using jModelTest, and the General Time Reversible (GTR) model with gamma-distributed rate variation was selected as the best-fit model based on Akaike Information Criterion. Phylogenetic trees were constructed using the maximum likelihood method in PhyML (http://phylogeny.lirmm.fr, accessed on February 15, 2025) with 1,000 bootstrap replications to assess the robustness of the tree topology. The final trees were visualized and annotated using FigTree v1.4.4. All RdRp sequences generated in this study were deposited in Genbank under accession numbers PV577202 to PV577312 for RdRp sequences, and PX070159 - PX070268 for 12S sequences.

### Statistical analyses

Statistical comparisons of coronavirus detection rates between environmental types (caves vs. temples) were performed using Fisher’s exact test of independence to assess the association between environment type and detection status while accounting for differences in sample sizes [[36,37]]. The test was chosen as appropriate for comparing proportions between two independent groups with categorical outcome data with a total sample size less than 1000. Statistical significance was set at α = 0.05. Statistical analyses were performed using Stata software (version 18).

## Results

### Samples collection and molecular screening of coronaviruses

A total of 648 bat guano samples were collected during the study period (December 2022 to June 2023), comprising 580 samples (89.5%) from bat roosting sites in the Than Pha Luang cave and 68 samples (10.5%) from the three temple sites (Vat Sisaket, Vat Phon Keng, and Vat Phosainyalam).

Population densities varied significantly across sampling sites. Tham Pha Luang cave exhibited the largest colony, with an estimated population of 100,000–1,000,000 individuals showing pronounced seasonal fluctuations (20% occupancy in February, 40% in April, and peak occupancy of 100% in June). In contrast, temple sites maintained smaller, more stable populations: VTESSK and VTEPNK each supported colonies of 50–100 individuals, while population size at VTEPHS could not be determined due to cryptic roosting behavior within palm tree foliage, though bat presence was confirmed through monk observations and guano collection. Lactating females and dependent pups were observed during the June sampling collection.

Molecular identification of bats species based on the amplification of 12S rRNA mitochondrial gene amplification revealed the presence of three distinct bat species across the sampling sites: *Hipposideros* spp. (*Hipposideridae* family, n = 100), *Taphozous* spp. (*Emballonuridae* family, n = 43), and *Chaerephon plicatus* (*Molossidae* family, n = 505) (Table 1).

**Table 1:** Number of samples collected according the sites and the species.

Bat species	Cave		Temple		Total	
	Positive (Rate%)	Tested	Positive (Rate %)	Tested	Positive (Rate %)	Tested
*Chaerephon plicatus*	105 (21.87)	480	3 (12)	25	108 (21.38)	505
*Hipposideros sp*	4 (4)	100	_	_	4 (4)	100
*Taphozous sp*	_	_	0 (0)	43	0 (0)	43
**Total**	**109 (18.8)**	**580**	**3 (4.41)**	**68**	**112 (17.28)**	**648**

The distribution of bat species showed clear habitat preferences. *Hipposideros* spp. were exclusively found in the cave environment, while *Taphozous* spp. were restricted to temple roosting sites. In contrast, *C. plicatus* demonstrated a broader distribution pattern, with the majority found in the cave but also present in the Vat Sisaket temple (VTESSK). This species distribution pattern suggests distinct ecological niches occupied by the different bat taxa within Vientiane province.

Beyond the three species confirmed through molecular analysis, field observations documented additional bat taxa across study sites. Cave sites other than the primary location exhibited greater taxonomic diversity: Pha Thor Nor Kham cave: *Aselliscus stoliczkanus* (~100 individuals) in deeper chambers and small groups of *Megaderma spasma* near the entrance; Tham Pha Luang Noi: *Hipposideros* sp. (~100 individuals) and *Rhinolophus* sp., though the latter species was absent during the June sampling period; Tham Pha Luang Middle: Multi-species assemblage comprising at least six species from four genera (*Rhinolophus*, *Megaderma*, *Hipposideros*, and *Glischropus*), with *Hipposideros armiger* representing the numerically dominant taxon.

### Coronavirus detection and prevalence

Of the 648 guano samples screened for coronavirus RNA using the pan-coronavirus RT-PCR approach, 112 samples tested positive, corresponding to an overall detection rate of 17.28%. A marked difference in coronavirus prevalence was observed between the natural and anthropized ecosystems. The cave environment showed significantly higher coronavirus detection rates (109/580, 18.8%) compared to the temple sites (3/68, 4.41%) (Fig 1, Table 1).

Species-specific analysis of coronavirus prevalence revealed substantial variation among the three bat species. *Chaerephon plicatus* exhibited the highest coronavirus positivity rate (108/505, 21.38%), while *Hipposideros* spp. showed a considerably lower detection rate (4/100, 4.00%). Notably, no coronaviruses were detected in any of the 43 samples from *Taphozous* spp. roosting in temples (Table 1). A statistically significant difference in coronavirus prevalence was observed between the natural and anthropized ecosystems. The cave environment showed significantly higher coronavirus detection rates (109/580, 18.8%) compared to the temple sites (3/68, 4.41%) (Fisher’s exact test value is 0.0019; p < 0.05).

### Phylogenetic analyses and genetic diversity of bat coronaviruses

**Fig 2:**
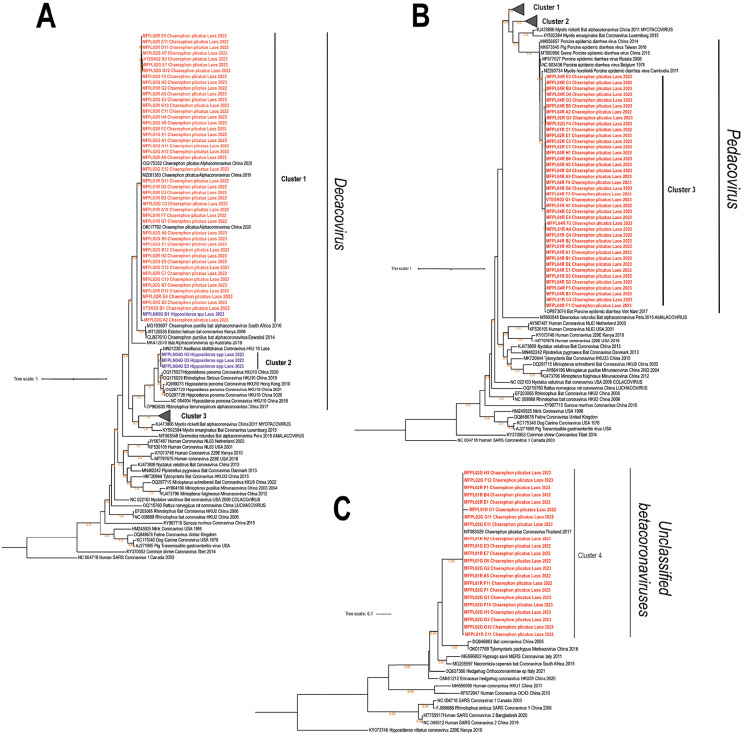
Phylogenetic tree based on a 410 bp fragment of the RNA-dependent RNA polymerase gene. Phylogenetic trees A and B represent the *Alphacoronavirus* genus, while phylogenetic tree C represents the *Betacoronavirus* genus. The GenBank accession number, host, country of origin, and sample collection year are indicated for all reference sequences. Bat-host coronaviruses are marked in red for *Chaerephon plicatus* and in purple for *Hipposideros* sp.

To characterize the genetic diversity of detected bat coronaviruses, we performed phylogenetic analysis on the 410 bp RdRp gene fragments obtained from positive samples. The phylogenetic tree (Fig 2) revealed that the bat coronaviruses in this study belonged to two distinct genera within the subfamily *Orthocoronavirinae*: *Alphacoronavirus* (90 sequences; 80.4%) and *Betacoronavirus* (22 sequences; 19.6%).

### Alphacoronavirus diversity

Phylogenetic analysis of the *Alphacoronavirus* genus revealed three distinct evolutionary lineages among our coronavirus sequences. The vast majority of *C. plicatus*-associated coronaviruses (82 sequences) formed a well-supported monophyletic group within the *Decacovirus* subgenus (Fig 2A), sharing 98–100% nucleotide identity with bat coronaviruses previously detected in *C. plicatus* from China [38–40]. Interestingly, one coronavirus sequence from *Hipposideros* spp. also clustered within this predominantly *C. plicatus*-associated clade, suggesting potential cross-species transmission or shared evolutionary history (Fig 2A).

A second distinct lineage within the *Decacovirus* subgenus comprised three coronavirus sequences from *Hipposideros* spp. that were closely related to Bat-CoV HKU10. These sequences formed a monophyletic group with previously characterized Bat-CoV HKU10 strains detected in various bat species including *Hipposideros pomona*, *Aselliscus stoliczkanus* (Hipposideridae), and *Rhinolophus sinicus* (Rhinolophidae), sharing 90–98% nucleotide identity (Fig 2A).

The third phylogenetic cluster differed markedly from the first two by grouping within the *Pedacovirus* subgenus. Forty coronavirus sequences from *C. plicatus* clustered with Porcine Epidemic Diarrhea Virus (PEDV). These sequences showed close relationship (92–93% nucleotide identity) to BtPEDV strain VNAB0123, a PEDV-related coronavirus previously detected in *C. plicatus* from Vietnam (accession number OP873074) (Fig 2B).

Notably, the three coronavirus sequences obtained from temple-roosting bats belonged exclusively to the Alphacoronavirus genus, with two sequences clustering in the *Decacovirus* subgenus (Cluster 1) and one sequence in the *Pedacovirus* subgenus (Cluster 3). The genetic profiles of these temple-derived coronaviruses were indistinguishable from those detected in cave-dwelling bats (Figs 2A and 2B).

### Betacoronavirus diversity

Twenty-two coronavirus sequences from *C. plicatus* clustered within the *Betacoronavirus* genus (Fig 2C). These sequences formed a monophyletic group within a clade of currently unclassified betacoronaviruses, showing close genetic relationship (97−100% nucleotide identity) to bat coronavirus PREDICT CoV-41 (accession number MT083529), previously identified in *C. plicatus* from Thailand.

Importantly, none of the betacoronaviruses detected in this study showed close genetic relationship to human-pathogenic coronaviruses, including those belonging to the *Merbecovirus* and *Sarbecovirus* subgenera, which contain MERS-CoV and SARS-CoV-1/2, respectively.

## Discussion

Bats play crucial ecological roles as pollinators and seed dispersers, yet they also serve as natural reservoirs for viruses of significant public health concern, including MERS-CoV and SARS-CoVs 1 and 2. These viruses have caused severe respiratory diseases that spread across multiple countries, demonstrating their epidemic and pandemic potential. The risk of bat coronavirus emergence is further amplified by their capacity for recombination, facilitated by overlapping host species ranges, co-roosting of different bat species, and the presence of multiple coronavirus strains within individual bats [9]. Understanding coronaviruses dynamics in bat populations is therefore essential for assessing the risk of potential spillover events that may lead to human-to-human transmission [41]. This study reports the detection of CoVs in guanos of bats species roosting in one cave and three temples in the Ventiane Province in Lao PDR.

### Coronavirus prevalence and ecological considerations

Our molecular screening revealed an overall coronavirus detection rate of 17.28%, which is notably higher than the 6.5% reported in a previous study investigating coronaviruses across various bat species and locations in Lao PDR [24]. This difference could be attributed to our focused sampling in habitats with high bat density, particularly the Than Pha Luang cave. Similar variations in positivity rates have been observed across Southeast Asia, with studies reporting rates of 7.2% in Thailand [26], 6.5–9.8% in Cambodia [19], and 4.7–10.2% in Vietnam [18]. Notably, McIver et al. (2020) detected coronaviruses predominantly in rodents rather than bats during their surveillance in Laos, highlighting the heterogeneity of results even within the same geographic area [25]. In southern China, which shares borders with Laos, Han et al. (2023) reported higher detection rates ranging from 14.7% to 26.1% in cave-dwelling bats, which align more closely with our findings, potentially reflecting similar ecological conditions [38]. Since the emergence of SARS-CoV-2 in 2019, surveillance efforts in Southeast Asia have intensified, focusing on bats and other wildlife. However, positivity rates from these investigations vary considerably across countries, limiting direct comparisons between studies. This variability is influenced by multiple factors, including bat species composition, geographic location, seasonal dynamics, and methodological approaches [42].

A remarkable finding of our study was the significant difference in coronavirus detection rates between cave (18.8%) and temple (4.41%) environments (Fisher’s exact test, p = 0.05). This disparity appears to run counter to established patterns in the literature regarding coronavirus prevalence and human land modification. Recent research has demonstrated that coronavirus prevalence typically increases with the intensity of human impact across various climates and biodiversity levels, with agriculture, deforestation, and mining being the most significant contributors to this pattern [43].

The unexpected lower prevalence in temple environments can be understood by examining the ecological characteristics of both habitats. Caves, such as Than Pha Luang in our study, represent natural environments that provide ideal conditions for sheltering large numbers of individuals and diverse bat species [44]. They offer permanent roosting sites with stable microclimatic conditions optimal for rearing young and maintain large colonies [45]. While Than Pha Luang is semi-anthropized—regularly exploited by local communities for guano collection and situated approximately 5 kilometers from agricultural landscapes including rice paddies and crop cultivation—it still maintains the fundamental ecological characteristics of a natural cave system.

In contrast, temple-roosting bats are synanthropic, cohabitating with humans in environments subject to various anthropogenic disturbances. Activities including tourism, artificial lighting, elevated sound levels, human movement, and prayer-related smoke can significantly impact bat abundance and behavior [46,47]. Several factors might explain the lower coronavirus prevalence in these temple environments. First, temples represent localized anthropogenic structures within largely preserved landscapes, rather than the wholesale ecosystem conversion associated with agriculture, deforestation, and mining that Warmuth et al. (2023) linked to increased coronavirus prevalence [43]. Second, specific human activities occurring in temples—religious ceremonies, tourism, maintenance—may create environmental conditions (such as incense smoke, regular cleaning, or altered microclimate) that are less conducive to coronavirus persistence than natural cave environments. Third, temple-roosting bat colonies may experience different population dynamics, with potentially lower densities or more transient occupancy than the more stable cave populations, which could influence viral transmission patterns. These preliminary findings suggest that the relationship between different forms of anthropization and viral ecology in temple environments warrants further investigation with larger sample sizes and broader geographic scope.

Among the three bat species identified in our study, *Chearephon plicatus* exhibited the highest coronavirus positivity (21.38%), followed by *Hipposideros* spp. (4%), while no coronaviruses were detected in samples from *Taphozous* spp. However, these rates should be interpreted with caution, as they may not reflect the true prevalence within each species. The molecular identification of the bat species revealed that most guano samples collected were from *C. plicatus*, potentially creating a sampling bias. Furthermore, although other bat species—including *Pipistrellus* spp., *Megaderma* spp., *Aselliscus* spp., and *Rhinolophus* spp.—were visually observed at the study sites, they were not molecularly identified in our samples. This absence may be attributed to our sampling strategy, which involved placing aluminum foils on accessible ground areas with minimal risk of environmental contamination. These accessible locations may predominantly harbor colonies of single bat species, potentially limiting the diversity captured in our sampling.

### Genetic diversity and host associations of detected coronaviruses

Phylogenetic analyses demonstrated that bats in our study harbored a diverse range of coronaviruses belonging to both *Alpha* and *Betacoronavirus* genera. Importantly, none of the detected coronaviruses in this study showed close genetically relationship to known human coronaviruses or belonged to the subgenera associated with human coronaviruses (*Duvinacovirus*, *Setracovirus*, *Embecovirus*, *Merbecovirus* and *Sarbecovirus)*, suggesting a relatively low zoonotic risk from the specific bat populations we studied. However, we identified alphacoronaviruses of the subgenera *Decacovirus* and *Pedacovirus*, as well as unclassified betacoronaviruses, which provide important insights into coronavirus diversity and evolution in this region.

### *Alphacoronavirus* genus

Within the *Decacovirus* subgenus, we observed two distinct clusters with apparent species specificity. Cluster 1, comprising primarily *C. plicatus*- associated coronaviruses from both cave and temple environments, were genetically close to coronavirus strains previously identified in *C. plicatus* in Yunnan province, China (98–100% nucleotide identity) [38–40]. Given that *C. plicatus* is widely distributed throughout Southeast Asia and southern China [48], and considering that Yunnan province shares a border with Lao PDR, these findings likely reflect intraspecific viral circulation facilitated by regional bat migration. This pattern suggests established viral lineages maintained within *C. plicatus* populations across geographical boundaries.

Intriguingly, we also detected evidence of potential cross-species transmission, as one coronavirus sequence from *Hipposideros* spp. clustered within the predominantly *C. plicatus*-associated Cluster 1. Since both species roost in the same cave, their close proximity in this confined habitat may facilitate viral transmission between species [49]. However, we cannot exclude the possibility of environmental cross-contamination among fecal samples collected from the cave floor, which represents an inherent limitation of non-invasive sampling approaches.

The second *Decacovirus* cluster (Cluster 2) comprised three coronavirus sequences from *Hipposideros* spp. that showed close genetic relationship to BtCoV-HKU10. This coronavirus was first identified in China in two bat species, *Hipposideros pomona* and *Rousettus leschenaultii* [50]. Initial analyses based on positive selection and molecular-clock calculation suggested that BtCoV-HKU10 likely resulted from a recent interspecies transmission from *R. leschenaultii* to *H. pomona* [50]. However, subsequent extensive coronaviruses surveillance in bats across Southeast Asia and Southwest China [24,26,51], has revealed greater genetic divergence and a broader geographic distribution of BtCoV-HKU10 in various *Hipposideros* bats hosts compared to *Rousettus* species, suggesting a longer independent evolutionary history in the former [52]. Our detection of BtCoV-HK10 from Laos aligns with the hypothesis that *Hipposideros* bats, particularly *H. pomona*, represent the natural reservoir of this coronavirus lineage [52] and confirms the circulation of BtCoV-HKU10 -related viruses in *Hipposideros* populations across Southeast Asia.

Our phylogenetic analyses also identified *C. plicatus* – associated coronaviruses belonging to the *Pedacovirus* subgenus (Cluster 3), which showed close genetic relationship (92–93% nucleotide identity) to strain VNAB0123 previously identified in *C. plicatus* from Cambodia [53]. This strain has been characterized as Porcine Enteric Diarrhea virus, indicating a wider geographic distribution of these viruses. PEDV, the prototype strain of *Pedacovirus* subgenus, poses significant public health and economic concerns as the causative agent of deadly watery diarrhea in piglets, representing a substantial threat to the swine industry [54]. Full genome analyses have suggested that PEDV strains likely evolved from bat alphacoronaviruses through cross species transmission to pigs [55]. However, the short RdRp fragment analyzed in our study is insufficient to conclusively determine whether our detected coronaviruses represent true PEDV strains or PEDV-related viruses. Further analyses based on the spike gene or complete genome sequences would provide more definitive characterization.

### *Betacoronavirus* genus

The betacoronaviruses identified in our study, exclusively from *C. plicatus* did not belong to any recognized subgenus within the *Betacoronavirus* genus. These unclassified betacoronaviruses were closely related (97−100% nucleotide identity) to bat coronavirus PREDICT CoV-41 previously identified in *C. plicatus* from Thailand. The detection of these unclassified strains underscores the incomplete understanding of bat coronavirus diversity and suggests that many novel coronaviruses remain to be discovered as sampling efforts intensify. Further characterization of these unclassified betacoronaviruses is warranted to assess their potential public health significance. We attempted whole-genome amplification for selected samples harboring these viruses; however, these efforts were unsuccessful, likely due to low viral loads or RNA degradation in the guano samples.

### Implications and future directions

This study provides valuable insights into coronavirus circulation among bats in Ventiane Province, Lao PDR, with a particular focus on temple environments—an understudied ecological niche at the human-wildlife interface. To our knowledge, this represents one of the first investigations specifically comparing coronavirus prevalence and diversity between temple-roosting and cave-dwelling bat populations in Southeast Asia. This study represents the first comparison of coronavirus detection rates between cave and temple bat roosts. While sample sizes differed between environments, the observed lower detection rates in temples (4.41%) versus caves (18.8%) warrant further investigation into how human-modified religious environments may affect viral ecology in bat populations.

Our phylogenetic analyses identified coronaviruses belonging to the *Decacovirus* and *Pedacovirus* subgenera of *Alphacoronavirus*, as well as unclassified betacoronaviruses. When integrated with previous findings, our results highlight the broad geographic distribution and host specificity of these viral lineages across Southeast Asia, while also revealing their presence in previously unexplored temple settings. Of particular significance is our detection of Cluster 3 (*Pedacovirus* subgenus) coronaviruses closely related to porcine epidemic diarrhea virus (PEDV). This finding represents a potentially important public health concern for several reasons. First, these viruses are circulating at considerable levels in bat populations, suggesting they are well-established and endemic. The homogeneous nature of this cluster indicates stable transmission within bat populations. Second, the close phylogenetic relationship between these bat-derived sequences and porcine coronaviruses provides compelling evidence of previous cross-species transmission events, demonstrating that these viruses have already overcome host specificity barriers. Third, the fact that these viruses have successfully established in both bats and pigs—a domesticated species in close contact with humans—highlights a potential pathway for eventual human infection. Fourth, in addition to coronaviruses that are strictly human-adapted, others have successfully crossed the species barrier, demonstrating their zoonotic potential. For instance, the detection of a recombinant canine-feline alphacoronavirus in pneumonia patients in Malaysia [56], and this highlights the need to enhance surveillance of alphacoronaviruses in domestic and farm animals that are in regular contact with humans. The zoonotic risk may be elevated precisely because these viruses already circulate in livestock populations that serve as potential intermediate hosts with frequent human contact. Our analysis revealed differential prevalence of PEDV-related coronaviruses across sampling sites, with an overall prevalence of 6.72% (39/580) in cave samples compared to just 1.47% (1/68) in temple environments. Specifically, the Vat Sisaket temple showed a prevalence of 2.5%, with one positive sample detected among the 40 guano samples collected at this site, while the other two temples yielded no PEDV-positive samples.

However, the relatively short gene fragment (440 bp of RdRp) used for screening limits comprehensive characterization of these viruses, emphasizing the need for full-genome analyses to better assess their zoonotic potential and evolutionary relationships. Longer genomic sequences, particularly of the spike protein gene, would provide critical information about receptor binding capabilities and host adaptation. The interface between humans, bats, and other animals represents a critical point for potential zoonotic disease transmission, with temples serving as unique settings where these interactions occur regularly through religious practices, tourism, and maintenance activities. In Lao PDR, human contact with wildlife, including bats, is common throughout the country through activities such as guano harvesting, cave exploration, and temple visitation [25]. These interactions, coupled with the genetic plasticity of coronaviruses and their demonstrated capacity for cross-species transmission, underscore the importance of continued surveillance and monitoring, particularly in these anthropized settings. Southeast Asia represents a recognized hotspot for the emergence of bat-borne zoonotic diseases, influenced by factors including high biodiversity, intensive land-use changes, and extensive wildlife trade. Understanding the ecology, diversity, and dynamics of bat coronaviruses in this region is therefore essential for developing effective strategies to prevent and mitigate future disease outbreaks. While our study detected no coronaviruses with clear zoonotic potential in the sampled bat populations, the identification of PEDV-related viruses that have previously demonstrated cross-species transmission capabilities highlights the potential role of bats in the evolution and maintenance of viruses with both economic significance and possible public health implications.

Future research should expand sampling to include additional bat species and temple locations throughout the region, as these anthropized environments remain largely unexplored in terms of their role in coronavirus ecology. Particular attention should be given to rhinolophid bats that have been implicated as reservoirs of SARS-related coronaviruses in northern Laos and neighboring regions [23]. Longitudinal surveillance across different seasons would provide valuable insights into temporal dynamics of coronavirus circulation, especially as human activity in temples may fluctuate with religious calendars and tourism patterns. Additionally, full-genome sequencing and functional characterization of identified viruses would enhance our understanding of their evolutionary relationships and potential for cross-species transmission. Combined with ecological studies and human behavioral surveys, these approaches would contribute to a more comprehensive understanding of the complex interactions between bats, their viruses, and human populations in Southeast Asia, particularly in these important but understudied anthropized religious settings.
